# Probiotics, Prebiotics and Other Dietary Supplements for Gut Microbiota Modulation in Celiac Disease Patients

**DOI:** 10.3390/nu12092674

**Published:** 2020-09-02

**Authors:** Giovanni Marasco, Giovanna Grazia Cirota, Benedetta Rossini, Lisa Lungaro, Anna Rita Di Biase, Antonio Colecchia, Umberto Volta, Roberto De Giorgio, Davide Festi, Giacomo Caio

**Affiliations:** 1 Department of Medical and Surgical Sciences (DIMEC), University of Bologna, Via Massarenti 9, 40138 Bologna, Italy; giovanna.cirota@gmail.com (G.G.C.); benedetta.rossini2@studio.unibo.it (B.R.); umberto.volta@unibo.it (U.V.); davide.festi@unibo.it (D.F.); 2 Department of Morphology, Surgery and Experimental Medicine, University of Ferrara, 44124 Ferrara, Italy; lisa.lungaro@gmail.com (L.L.); dgrrrt@unife.it (R.D.G.); 3 Department of Pediatrics, Policlinic Hospital, University of Modena, Via del Pozzo 71, 41126 Modena, Italy; dibiase.annarita@gmail.com; 4 Gastroenterology Unit, Borgo Trento University Hospital of Verona, P.le Aristide Stefani 1, 37126 Verona, Italy; antonio.colecchia@aovr.veneto.it; 5 Celiac Center and Mucosal Immunology and Biology Research, Massachusetts General Hospital-Harvard Medical School, Boston, MA 02114, USA

**Keywords:** celiac disease, CD, gut microbiota, probiotics, prebiotics, oat

## Abstract

To date, the only available treatment for celiac disease (CD) patients is a life-lasting gluten-free diet (GFD). Lack of adherence to the GFD leads to a significant risk of adverse health consequences. Food cross-contamination, nutritional imbalances, and persistent gastrointestinal symptoms are the main concerns related to GFD. Moreover, despite rigid compliance to GFD, patients struggle in achieving a full restoring of the gut microbiota, which plays a role in the nutritive compounds processing, and absorption. Pivotal studies on the supplementation of GFD with probiotics, such as *Bifidobacterium* and *Lactobacilli*, reported a potential to restore gut microbiota composition and to pre-digest gluten in the intestinal lumen, reducing the inflammation associated with gluten intake, the intestinal permeability, and the cytokine and antibody production. These findings could explain an improvement in symptoms and quality of life in patients treated with GFD and probiotics. On the other hand, the inclusion of prebiotics in GFD could also be easy to administer and cost-effective as an adjunctive treatment for CD, having the power to stimulate the growth of potentially health-promoting bacteria strains. However, evidence regarding the use of prebiotics and probiotics in patients with CD is still insufficient to justify their use in clinical practice.

## 1. Introduction

Celiac disease (CD) is a common systemic disorder mainly affecting the small intestine [1], due to the abnormal response of human immunity to gluten ingestion. CD onset is favored in subjects carrying genetic susceptibility (HLA-DQ2/DQ8 positivity and non-HLA genes), under the influence of triggering environmental factors, such as viral infections and dysbiosis of the gut microbiota [2]. Although 30–40% of the global population carries the HLA DQ2/DQ8 genotype, only 1–1.5% of them express the CD phenotype, meaning that other factors, such as diet and environment, take part in the illness outbreak [2,3]. The term “gluten” comprises more than 100 ethanol-soluble proteins, (i.e., prolamins and glutelins), derived from wheat, rye, and barley. These proteins show specific features, such as hydrophobicity and repeated amino-acid sequences rich in glutamine and proline residues, which enhance protein resistance to human intestinal proteases digestion [2,3,4]. To date, the unique treatment that has proven to be effective in CD patients is strictly life-long adherence to the gluten-free diet (GFD), i.e., a diet containing less than 10 mg per day of gluten [1,2]. Lack of adherence to the GFD leads to a significant risk of adverse health consequences [5] and increases mortality from malignancies (e.g., small bowel adenocarcinoma, cancer of the esophagus, B-cell and T-cell non-Hodgkin lymphomas), and in particular intestinal T-cell lymphomas; the risk reduces with strict adherence to the diet [6,7]. Despite rigid compliance to GFD, several studies showed that patients struggle in achieving a full restoring of the gut microbiota; the cause of this phenomenon could be inferred from a persistent genetic influence than to the lack of prebiotics usually ingested with gluten assumption [8]. The supplementation of GFD with probiotics, such as *Bifidobacterium* and *Lactobacilli*, could help to restore altered gut microbiota, reducing both gliadin toxicity and immune activation [9], while improving the daily ingestible gluten amount to better tolerate the GFD. Besides, considering the alterations of the gut microbiota as an environmental factor promoting the CD pathogenesis, and considering that the genetic background may influence its composition in subjects at high risk, probiotics administration may have a role in primary prevention for subjects at high risk for CD [10]. Today, research is seeking new nutritional possibilities to improve the life of CD patients, suggesting innovative therapeutic approaches, such as supplements able to reduce intestinal permeability or to suppress the inflammatory immune response, such as probiotics and prebiotics [11]. Thus, this review aims to summarize the recent advances in probiotics, prebiotics, and other dietary supplements for gut microbiota modulation in CD patients.

## 2. Impact of the Gluten-Free Diet on the Microbiome

The only effective treatment for CD is a life-long strict observance of GFD, which means not only avoiding gluten-containing foods but also gluten contaminations [12]. Among cereals, gluten is present in durum wheat, bread wheat, barley, rye, Khorasan wheat, the three species of spelt, and triticale; other gluten-containing wheat derivates are bulgur and seitan. Gluten has a low nutritional value, but it confers important qualities to foods, improving palatability; it is almost ubiquitous in refined foods that are not declared gluten-free (GF), and that thereby should be avoided by celiac patients [13]. Although the range of high-quality GF products has increased in recent years, and the GFD has gained broad acceptance, it still shows many downsides [13]. First of all, the major issue of GFD is the gluten-free foods’ cross-contamination, which can occur both in the production lines or during the preparation of gluten-free foods at home or when eating out [12]. For this reason, it is suggested to provide CD patients with the knowledge and the skills to adhere to a correct GFD, advising them about “how to read” food labels [14]. Secondly, the GFD is not a complete and balanced diet, but it leads to micro- and macronutrients deficiencies, which is of particular importance when affecting children’s metabolism. In particular, gluten-free foods lack minerals (calcium, iron, magnesium, and zinc), vitamins (vitamin B12, folate, and vitamin D), and fibers [15]. Some authors propose dietary education for CD patients to promote “nutritional awareness”, as many of the inadequacies of dietary intakes, such as the insufficient intake of fiber and folate, may depend on individual food choices. In contrast, some deficiencies, such as thiamin, are CD specific [16]. Moreover, GFD is also a threat to health because it increases dietary exposure to arsenic and other toxic contaminants [17]. Finally, CD patients are nowadays exposed to nutritional imbalances by assuming foods that are high in sugars, fats, and calories, or rich in proteins, like eggs and meat, in addition to snacks with a high content of lipids and low fibers [18,19], which may additionally lead to irritable bowel syndrome-like symptoms [20]. Indeed, the majority of gluten-free commercial grain-based products contain less fiber than their gluten-containing equivalents so patients may report weight gain and constipation [21].

GFD induces microbial shifts with possible consequent impairment of the immune-metabolic homeostasis, which may contribute to functional symptoms’ persistence. Only a few studies longitudinally evaluated gut microbiota before and after long-term GFD. Pivotal studies on duodenal mucosal and fecal microbiota showed an incomplete restoration of the microbial composition after two years of GFD [22,23], with persistently low levels of *Bifidobacteria* and *Lactobacilli* along with reduced bacterial diversity [24,25]. Persistently impaired microbial shifts were subsequently found by the same research group [24] focused on *Bifidobacterial* strains and by another research group [25]. Di Cagno et al. [26] found that CD children treated with GFD had lower levels of *Lactobacillus*, *Enterococcus*, and *Bifidobacteria*, and increased levels of *Bacteroides*, *Staphylococcus*, *Salmonella*, *Shigella*, and *Klebsiella* in comparison to healthy controls. Moreover, CD children on a GFD showed lower levels of short-chain fatty acids (SCFAs) with respect to those patients on the gluten-containing diet. Thus, it is possible to conclude that an incomplete microbial restoration is present in CD patients despite the instauration of a GFD. This finding is not surprising since a previous study [27] on healthy subjects undergoing GFD reported a decrease in *Bifidobacterium*, *Bifidobacterium longum*, *Clostridium lituseburense*, *Lactobacillus*, and *Faecalibacterium prausnitzii*, and an increase in *Enterobacteriaceae* and *E. coli* strains. Moreover, the inflammatory pathway was consistently imbalanced due to GFD, with a proinflammatory shift. This shift might be explained by the reduction in prebiotics included in the gluten-containing diet [28]. Longitudinal evaluation in the same patients before and after GFD confirmed these results, reporting reduced abundances of *Lactobacillus*, *Bifidobacterium*, and *Bacteroides* [29]. Similar results were also reported in a small study on children [30]. Besides a significant impairment in gut microbial restoration characterized by reduced bacterial richness, reduced levels of *Bacteroidetes* and *Firmicutes* and higher levels of *Proteobacteria* have also been found after three years of GFD in CD patients complaining of irritable bowel syndrome-like symptoms [20]. Notably, dysbiosis, namely a microbial imbalance, is also linked to several conditions among which irritable bowel syndrome (IBS) is one of the most common and, as CD, is characterized by different gastrointestinal symptoms. In this setting, several probiotic strains, such as *L. plantarum* PBS067, *B. lactis* BL050, *L. acidophilus* PBS066, *L. rhamnosus* LRH020, and *L. reuteri* PBS072, have been demonstrated to improve gastrointestinal distresses thanks to gut eubiosis recovery [31,32].

## 3. Methods

This narrative review aims to describe the effects of probiotics, prebiotics, and other dietary supplements on the gut microbiota of CD patients in GFD. In the preparation of this manuscript, we followed the narrative review checklist by the Academy of Nutrition and Dietetics. We conducted a PubMed, MEDLINE, and Scopus search from inception to March 2020 using the search terms ‘gluten’ AND ‘celiac disease’ AND ‘microbiota’ AND ‘gluten-free diet’ OR ‘probiotics’ OR ‘prebiotics’ followed by a manual review of the literature to select relevant articles for this clinical review. Literature researches were carried out based on title and abstract; the reference list of each relevant article was evaluated in order to find any other relevant articles. Randomized controlled trials, cross-sectional studies, and eminent reviews on the topic were included in the present narrative review if they reported data on the microbiota composition or metabolomic of celiac disease patients during GFD or probiotic or prebiotic or other dietary compound supplementation. We restricted the search strategy for language to English articles. The article search was carried out independently by two authors (GM and GGC).

## 4. Dietary Supplements Beyond the Gluten-Free Diet

Despite strict GFD adherence, a certain degree of mucosal inflammation and symptoms may persist in up to 5% of CD patients. Non-responsive CD (NRCD), unlike refractory CD (RCD), has a higher prevalence, affecting from 7% to 30% of celiac patients [33,34]. Therefore, a supportive pharmacological treatment would be suggested [16,17,35,36,37]. Besides, several researchers focused their studies on finding additional dietary compounds able to increase gluten tolerability; among these, prebiotics and probiotics play a leading role.

### 4.1. Probiotics

The evidence of dysbiosis in CD patients [38,39,40] has gained more and more research on the use of probiotics for gut microbiota restoration and modulation. Indeed, the composition of gut microbiota influences the spectrum of gastrointestinal symptoms of this disease [40]; microbiological studies [26,41,42] showed a different abundance of *Lactobacillus* and *Bifidobacterium* strains in CD patients at disease diagnosis, other than a reduction in several ‘health’-promoting bacterial strains, such as *Akkermansia muciniphila*, demonstrating an association with intestinal dysbiosis [43]. The unbalanced gut microbiota may indeed promote CD, influencing the mutualistic relationship between the colonic microbiota, their metabolic products, and the host immune system; in order to maintain immunological homeostasis, it is essential to establish a “healthy relationship” since the first years of life [44]. Supporting these hypotheses, a study by Wacklin et al. [38] showed that intestinal dysbiosis is linked with refractory gastrointestinal symptoms, iron deficiency, low bone density, and anemia in CD patients on GFD. As a matter of fact, CD is strongly influenced by dysbiosis, and several theories postulate that it could facilitate a loss of gluten tolerance in genetically predisposed subjects, increasing the gut mucosal permeability, with the leakage of tight junctions during inflammation and the recruitment of T cells [41,45,46,47].

Probiotics are defined according to the World Health Organization as live microorganisms, which, if administered in adequate amounts, can confer health benefits to the host [48]. Probiotics use in CD could modulate the composition and functions of the microbiota; this may delay the onset of the disease or prevent it. Probiotics are also able to regulate the immune response, the degradation of toxin receptors, the competition for nutrients, the blockage of adhesion sites, and the production of inhibitory substances against pathogens [49]. Studies evaluating the effect of probiotics in CD patients are summarized in Table 1 and Table 2 [8,50,51,52,53,54,55,56,57,58,59,60,61,62,63,64,65,66]. In particular, a study by Lindfors et al. [50] highlighted that specific probiotics, such as *Lactobacillus fermentum* or *Bifidobacterium lactis*, have a protective role against the toxic effects of gliadin in intestinal cell cultures (human colon Caco-2 cells), with the result of dose-dependent inhibition of increased epithelial gliadin-induced permeability and stimulation of IL-10 production by regulatory T-cells [55,67]. Indeed, the activation of inflammation through induction of the cytokines cascade by the NFkB pathway is one of the leading causes of symptoms. Other *Bifidobacterial* strains are able to improve the altered gut bacterial composition in CD, reducing inflammation, as demonstrated by Laparra et al. [68]. In another study, the same group found that gliadin-digested fragments and *Bifidobacteria* (specifically *B. longum* CECT 7347) induce a downregulation of the mRNA expression of proinflammatory cytokines (NFkB, TNF-alpha, and IL-1beta) [52]. The same species were also able to restore the expression of liver transferrin receptor (TfR)-2, lowered by gliadin, and to improve gliadin-mediated perturbations, such as liver iron deposition and mobilization [69]. Moreover, *Lactobacillus casei* seemed to be useful in CD for the recovery of gut-associated lymphoid tissue (GALT) homeostasis and a healthy mucosal structure [51].

**Figure 1:**
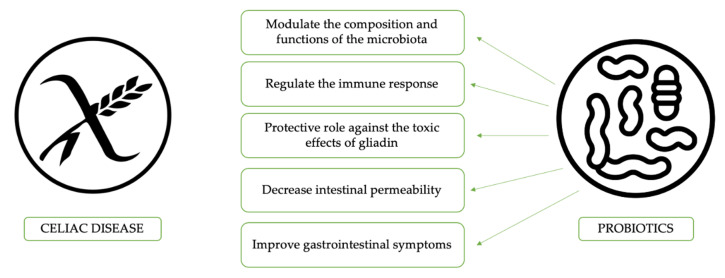
The potential benefits of probiotics use in celiac disease patients.

Probiotics effects in the specific setting of CD patients are summarized in Figure 1. To understand the role of some bacterial clusters in the modulation of the immune response, the effect of *Bifidobacterium bifidum* and *Bifidobacterium longum* on peripheral blood mononuclear cells, alone or with CD triggers, were compared to Gram-negative bacteria, such as *Bacteroides fragilis* and *Escherichia coli*. It was found that Gram-negative bacteria induce a higher secretion of TH-1 proinflammatory cytokines and activation mechanisms (HLA-DR, CD40, IL-12, and IFN-c) with respect to the *Bifidobacterium* strains [70]. The latter strain, on the other hand, was able to upregulate CD83 expression, which is a marker of mature dendritic cells. Thus, the authors concluded that microbiota could regulate monocytes and the IFN reaction to gliadin locally. Other common topics among the studies about probiotics and the amelioration of intestinal symptoms in celiac patients is the impact of “good” bacteria on the microbiota. Previous studies using VSL#3 in CD showed that *Streptococcus thermophilus*, *Lactobacillus plantarum*, *Lactobacillus acidophilus*, *Lactobacillus casei*, *Lactobacillus delbrueckii* spp. *Bulgaricus*, *Bifidobacterium breve*, *Bifidobacterium longum*, and *Bifidobacterium infantis* were able to decrease the toxicity of wheat flour during long-term fermentation, due to complete hydrolysis of the gliadin [54]. Additionally, a reduced reorganization of intracellular F-actin with decreased release of zonulin led to decreased intestinal permeability [54]. Conversely, Smecuol et al. [56] studied the effect of *Bifidobacterium infantis natren life start* strain administration on CD patients on a gluten-containing diet, observing an increase of the intestinal permeability. It is possible that probiotic administration did not modify the intestinal permeability probably due to an insufficient dose or to the short time of administration; however, it improved gastrointestinal symptoms, mainly referred to as better digestion and reduced constipation. A similar study evaluated the effects of 3 months of GFD supplemented with *Bifidobacterium longum* CECT 7347 in newly diagnosed CD children, showing a decrease in CD3 T-cells, a reduction in the *Bacteroides fragilis* group and in the content of IgA in stools, and, eventually, an improvement of the symptoms in CD patients [57]. As a confirmation of probiotics’ effect on CD symptoms, a study carried out in Argentina identified significant alterations in the amount of *Lactobacillus* strains in symptom-free CD children; five different *Lactobacilli* were isolated in the stools of healthy children and *Lactobacillus rhamnosus* and *Lactobacillus paracasei* were proposed as potential probiotic strains since they show high resistance to gastrointestinal tract conditions [58].

In conclusion, *Bifidobacteria* and *Lactobacilli* administration seems to have the potential to restore gut microbiota composition and to predigest gluten in the intestinal lumen, reducing the inflammation associated with gluten intake, intestinal permeability, and cytokine and antibody production. These findings could explain an improvement in symptoms and quality of life in patients treated with GFD and probiotics. However, since evidence on this topic is still scarce, routine use in the clinical practice of probiotics is still not advised by international guidelines for the use of probiotics [71].

### 4.2. Prebiotics

Prebiotics are defined according to the International Scientific Association for Probiotics and Prebiotics (ISAPP) consensus statement as a substrate that is selectively utilized by host microorganisms conferring a health benefit [72]. Among the new therapies recently proposed, prebiotics are a promising and safe additive to GFD with a beneficial influence on human health [73]. Prebiotics have the power to stimulate the growth and activity of potentially health-promoting bacteria strains in the intestine, mainly *Bifidobacterium* and *Lactobacillus*. For this reason, their ability to regulate the activity of gut microbiota could be used to address CD-related symptoms. Literature data lead to the hypothesis that the inclusion of prebiotics in GFD could also be easy to administer and cost-effective as an adjunctive treatment for CD [73]. Only a few pilot human studies have been carried out to clarify the impact of prebiotics on the intestinal inflammation in general and, in particular, on CD [43,56]; most of them include formulations of prebiotics combined with other ingredients. Krupa-Kozak et al. [43] carried out one of the first studies on this subject: a randomized placebo-controlled clinical trial aiming to assess the influence of an oligofructose-enriched inulin, named “Orafti^®^-Synergy1”(Tienen, Belgium), on pediatric CD patients following GFD. The authors found an increase in the count of *Bifidobacterium* and a decrease of *Lactobacillus* in the pediatric population analyzed. This result was different from CD patients assuming the placebo, in which a decrease in the count and diversity of *Bifidobacterium* species was reported. Indeed, *Bifidobacterium* can prevent entomopathogen infections and reduce gastrointestinal mucosal inflammation [74,75]. In parallel, Adebola et al. [46] demonstrated that inulin is not able to directly stimulate any of the five probiotic strains of *Lactobacillus*, whereas other potential prebiotics, including lactulose and lactobionic acid, may exert this effect and represent an optimal substrate for bacteria to minimize the adverse effects of bile acid stress. Indeed, these authors found that *Lactobacillus* strains, in particular *Lactobacillus acidophilus* NCFM and *Lactobacillus reuteri* NCIMB 11951, have stimulating effects only in specific formulations of probiotics and prebiotics with lactulose or lactobionic acid [46]. A similar study by Tuohy et al. [76] observed a significant increase in the *Bifidobacterium* count in healthy volunteers receiving inulin for two weeks. Furthermore, another important study [43] showed that the addition of oligofructose-enriched inulin to GFD improved the fecal microbiota, increasing the total SCFAs, such as propionate and butyrate, remarkably. This effect could be attributed to the fermentation of inulin-type fructans (ITFs) as a readily available source of energy for gut microbiota present on the prebiotic formula, which is usually a mixture of short-chain fructo-oligosaccharides and long-chain inulin. In particular, butyric acid is the primary fermentation product of oligosaccharides, whereas inulin fermentation leads to the formation of propionic acid; the latter has a role in the proliferation and differentiation of colon epithelial cells (tight-junction proteins, claudin-1, zonula-occludens-2) [77,78]. Thus, supplementation with “Orafti^®^-Synergy1” could be a promising therapeutic approach for modulating intestinal microbiota and it could also have future employment in other autoimmune diseases like diabetes type I, considering the putative role of intestinal microbiota in their development [79,80]. Drabisnka et al. [80,81,82] conducted additional studies on the utility of “Orafti^®^-Synergy1” and its effects on CD children’s metabolism and microbiota. They reported that “Orafti^®^-Synergy1” had an effect on iron homeostasis in CD patients treated with GFD, bringing about a significant decrease in the plasma hepcidin concentration, which is a key regulator for duodenal iron, resulting in a positive effect on its absorption. Moreover, since the risk of an adverse calcium balance and reduced bone density in CD patients is induced both by the disease and by the GFD, they assessed the effects of gluten-free bread with additional calcium (Ca) in a rat model [83]. As a result, they found that the dietary inulin in GFD influences the intestinal microbiota positively by the stimulation of Ca absorption, especially in conditions of Ca malnutrition. They concluded that the addition of inulin to a GFD could be a promising strategy for beneficial modulation of intestinal microbiota and the improvement of Ca absorption. The same authors [84] evaluated, through an in vitro study on Caco-2 cells treated with a human intestinal bacteria suspension, whether inulin and fructo-oligosaccharides (FOSs) can influence Ca uptake. They found that the addition of ITF might enhance cellular Ca uptake by altering the rate and amount of organic acids, such as butyric, valeric, and lactic acids (produced by the intestinal microbiota) and stimulating cellular Ca retention, reducing its transport. In another study, Capriles et al. [85] tried to investigate the impact of ITFs, added in different quantities (0%, 4%, 8%, 10%, and 12%) to GFD, on the sensory and nutritional quality of the diet. Indeed, ITFs can cause gas retention during baking, improving GFD quality by yielding a better specific volume, softer crumb, and improved crust and crumb browning with ameliorated sensory acceptance. The addition of 12% ITFs to the standard formulation is required in order to obtain GFD enriched with 8% ITFs (4 g of fructans per 50-g bread serving size), and can provide health benefits: Decrease of the glycemic index (from 71 to 48) and glycemic load (from 12 to 8) and improved Ca absorption. The results of this study are in line with those reported by Korus et al. [86] describing the partial replacement of starch in GFD recipes for 3.5% and 8% inulin. The effects of prebiotics on gut microbiota have also been elucidated by a crossover study by Fuller et al. [87], who tried to enhance the concentration of colonic *Bifidobacterial* populations with the use of a commercially available prebiotic (inulin). They found that after 16 days of treatment, the fecal *Bifidobacterial* population was significantly higher. In conclusion, prebiotic use in CD patients has largely not been investigated and their use, mainly that of inulin, has been proved to enhance the abundances of ‘beneficial’ bacterial strains, such as *Bifidobacterium* and *Lactobacillus* strains, other than enhancing the homeostatic and metabolic activity of these strains. However, to date, sufficient data are not currently available to definitely recommend their use in the routine clinical practice.

### 4.3. Synbiotic in Celiac Disease

The growth of probiotics in the large bowel could be improved through the synergistic combination with prebiotics; for this purpose, it has been created the term “synbiotic”, which defines a product in which a prebiotic is specifically added to favour the growth of the wanted probiotic [88]. As an example, a study by Furrie et al. [47] developed a synbiotic for patients with ulcerative colitis (UC). It combines a probiotic, the Bifidobacterium longum isolated from healthy rectal epithelium, with the prebiotic “Orafti^®^-Synergy1” (a preferential inulin-oligofructose growth substrate for the probiotic strain), achieving proper compliance and symptoms improvement in the acute phase [47]. Moreover, a study by Adebola et al. Ref. [46] examined the ability of three potential prebiotics, inulin, lactulose and lactobionic acid to support the growth of five probiotics lactobacilli cultures and provide protection from bile acid stress. Of the five tested probiotics, only *Lactobacillus acidophilus* NCFM and *Lactobacillus reuteri* NCIMB 11951 utilized lactulose. Similar variability was observed with the ability of the prebiotics to protect probiotics from bile acid stress: both *Lactobacillus acidophilus* NCFM and *Lactobacillus reuteri* NCIMB 11951 were able to grow in 2 mM cholic and taurocholic acid when incubated in synbiotic combinations with lactulose (1%) or lactobionic acid (1%). Although synbiotic preparations are increasingly used, the potential benefits to gut health may be limited, as only specific combinations may enhance probiotics survival and growth. For this reason, nowadays, prebiotics are increasingly added to probiotic food preparations (synbiotic) to enhance probiotics survival and growth [46]. However, in the specific CD setting, no studies are available exploring the combined effect of probiotics and prebiotics.

### 4.4. Other Dietary Supplements

Recent studies showed that diet and dietary compounds are playing an increasing role in the modulation of the metabolic activity of the intestinal microbiota. As example beyond CD, the randomized trial by Beaumont et al. [89] in overweight patients analyzed the effects of a high-protein diet (HPD) on gut microbiota composition, metabolic activity, and large intestine mucosal gene expression. The authors reported that a three-week administration of HPD was sufficient to alter the bacterial metabolite production and to modify gene expression in the rectal mucosa. These findings could be explained as a consequence of the increased level of the amino acid-derived bacterial metabolites’ concentration and of the decreased butyrate production, two factors leading to the modification of several key homeostatic processes at the gene expression level in the rectal mucosa. On the other hand, according to the experience by Wu et al. [90] comparing the metabolomic activity of two groups of subjects with agrarian or ‘Westernized’ diet, dietary delivery of substrates to the gut microbiota is not enough for the control of the production of metabolites, which needs the coexistence of specific bacterial lineages [90]. As a matter of fact, focusing on CD patients, a recent systematic review by Rondanelli et al. [91] underlined that in CD patients on a long-term gluten-free diet with good compliance, a micronutrient deficiency was detected in up to 30% of subjects for vitamin B12, 40% for iron and zinc, 20% for folic acid and in children for magnesium, and 25% for vitamin D; thus, the authors suggested that dietary supplementation of these micronutrients would be useful. This evidence may be explained by several factors, such as the GFD itself, the unbalance of GFD on a more ‘agrarian’ or ‘Westernized’ pattern, and the residual dysbiosis of CD patients which together obstacles the presence of specific bacterial lineages able to guarantee an adequate production of metabolites and absorption of micronutrients. However, a recent systematic review by Melini et al. [92] found that in the last decade, GF products are increasingly richer in fiber content, which may be helpful in the shaping of an adequate microbial profile of these patients.

## 5. Impact of Oat Intake

Another dietary controversy is the inclusion of oats in the diet of CD patients. Oats are a great source of nutrients, often lacking in the gluten-free diet, such as iron and fiber; however, several studies have shown the possibility of cross-reactivity: The avenin (a protein similar in function to gluten) in oats can activate gluten-reactive T cells [93]. A randomized double-blind multicenter study [94] focused on the effect of a GFD containing oats (GFD-oats) compared to a standard GFD (GFD-std) in 116 children (GFD-oats, *n =* 57; GFD-std, *n =* 59) with newly diagnosed CD. The authors found a significant decrease in the total SCFA concentration in the GFD-std group during a year of GFD but not in the GFD-oats group, in which the proinflammatory acetic acid and total SCFA concentration remained high throughout the diet period. An explanation could be the higher fiber content, due to the addition of oats to the GFD, thus providing more substrate for fermentation. Lundin et al. [95] raised some concerns regarding the safety of oats in adults with CD; in fact, they concluded that several CD adults exposed to oats in their GFD appear to experience abdominal symptoms or even produce avenin-reactive T-cells in the small intestinal mucosa [96]. On the other hand, Koskinen et al. [97] reported that oats did not induce transglutaminase-2 autoantibody production at the intestinal mucosal level in CD children over a 2-year follow-up [97]. In the groups analyzed, CD children had similar serum anti-avenin antibody titers following 1-year GFD-std or GFD-oats treatment, but some of the CD children in both study groups continued to excrete high amounts of urinary nitrite/nitrate, an indicator of upregulation of inducible nitric oxide synthase, after proinflammatory cytokine stimuli [98,99]. Considering the positive nutritional effect of oats, further studies are needed to deeply evaluate the use of this cereal, which, however, needs to be strictly wheat free [100].

## 6. Conclusions

Different dietary and supplementary strategies are being explored, aiming to support the compliance and the response of CD patients to GFD and to avoid complications and severe progression of the disease. The synergic use of prebiotics, together with prebiotics, could be a promising therapeutic approach for modulating intestinal microbiota composition and function. Nevertheless, evidence regarding the use of prebiotics and probiotics in patients with CD is still insufficient to justify their use in routine clinical practice. Well-designed randomized controlled trials are needed to clarify their role in CD patients.

## Figures and Tables

**Table 1:** Studies evaluating probiotic use in celiac disease in vitro, ex vivo, and in animal models.

Author, Year	Composition, Strains	Duration of Administration	Study Design	Aims and Findings	Meaning
Lindfors K. et al., 2008 [50].	*Bifidobacterium lactis*	In vitro study	-	Inhibit the gluten/gliadin-induced damage in the small-intestinal mucosa.	Inhibition dose-dependent to increased epithelial gliadin-induced permeability and stimulation of IL-10 production by regulatory T-cells.
D’Arienzo et al., 2011 [51].	*Lactobacillus casei* ATCC 9595	35 days	Animal study	Complete recovery of villous blunting, decreased weight loss and recovered basal TNF-α levels.	*L. casei* was effective in rescuing the normal mucosal architecture and Gut associated lymphoid tissue homeostasis.
Laparra et al., 2012 [52].	*Bifidobacterium longum* CECT 7347	10 days from birth	Animal study	In gluten-sensitized animals *B. longum* administration increased NFκB expression, IL-10, CD8+, but reduced TNF-α expression, CD4+ and CD4+/Fox3+ cell populations.	*B. longum* regulates inflammatory cytokine production and CD4+ T cell mediated immune response in an animal model of gliadin induced enteropathy.
Papista et al., 2012 [53].	*Saccharomyces boulardi* KK1 strain, hydrolyzed the 28-kDa gliadin fraction	30 days	Animal study	*S. boulardi* administration improved enteropathy development, decreased epithelial cell expression of CD71 and localized cytokine production.	A new mouse model for human CD based on histopathological features and common biomarkers. *S. boulardi* showed activity in the treatment of CD by reversing disease development.

Celiac disease (CD), Tumor Necrosis Factor alpha (TNF-α).

**Table 2:** Studies evaluating probiotic use in celiac disease patients.

Author, Year	Composition, Strains	Duration of Administration	Study Design	Number of Participants	Aims and Findings	Meaning
De Angelis et al., 2006 [54].	VSL#3	-	Comparative study	-	VSL#3 can largely colonize the intestine for a long period.	VSL#3 treatment would eliminate any traces of toxic peptides in processed foods minimizing the long-term risks and improving the quality life.
Medina M. et al., 2007 [55].	*Bifidobacterium longum*	4 months	Comparative study	-	Genomic DNA of some strains stimulated the production of Th1 and pro-inflammatory cytokines, interferon-gamma and TNF-a, instead of IL-10.	Immunomodulatory activity of *B. longum.*
Smecuol et al., 2013 [56].	*Bifidobacterium natren life start*	3 weeks treatment, follow up on day 50	Double blind, randomize, placebo-controlled trial	22 (*n =* 12 *B. NLS*, *n =* 10 placebo)	Effect on intestinal permeability; outcome of clinical symptoms by GSRS questionnaire; modification of immunologic indicators influenced by gluten.	Administration of *Bifidobacterium NLS* to untreated CD patients does not modify protein abnormalities but might improve symptoms and produce immunologic changes.
Olivares et al., 2014 [57].	*Bifidobacterium longum* CECT 7347	3 months	Double blind, randomized, placebo-controlled trial	33 (*n =* 17 *B. longum* CECT 7347, *n =* 16 placebo)	Baseline and post-intervention outcomes (immune phenotype of peripheral blood cells, serum cytokine, fecal secretory IgA, anthropometric parameters and intestinal microbiota composition).	Patients undergoing probiotic treatment showed greater height percentile, decreased peripheral CD3+ T lymphocytes, and slightly reduced TNF-α concentration. Additionally, reduced B. fragilis and secretory IgA in the stool.
Pisarello et al., 2014 [58].	*Lactobacillus rhamnosus; Lactobacillus paracasei*	11 months	Comparative study	30 (*n =* 15 healthy, *n =* 15 CD children)	*Lactobacillus* counts in the CD children on a GFD group revealed significantly lower values than those in the healthy controls group.	Treatment with probiotics cannot replace GFD but is able to attenuate the altered inflammatory parameters in celiac individuals and to modify the composition of the intestinal microbiota.
Golfetto et al., 2014 [59].	*Bifidobacteria* spp.	-	Comparative study	14 CD patients, 42 health control	The concentration of *Bifidobacteria* per gram of feces was significantly higher in healthy subjects (controls) (1.5 ± 0.63 × 108 CFU/g) when compared to celiac patients (2.5 ± 1.5 × 107 CFU/g).	Lower levels of *Bifidobacterial* can provide an imbalance in the intestinal microbiota of CD patients, regardless of pH, even while on a gluten-free diet.
Klemenak et al., 2015 [60].	*Bifidobacterium breve* BRO3 and *B. breve* B632	3 months	Double-blinded, placebo-controlled trial	49 CD children (*n =* 24 *B. breve* BRO3 and *B. breve* B632, *n =* 25 placebo), 18 healthy control	Outcomes: level of Serum production of IL-10; TNF-α.	TNF-α levels decreased after 3 months of probiotic treatment, however on follow up after 3 months, the levels increased. The IL-10 levels were below detection.
Quagliariello et al., 2016 [61].	*Bifidbacterium breve* strains B632 and BRO3	3 months	Double-blinded, placebo-controlled study	40 CD children, 16 healthy control	Determination of microbiome after probiotic treatment.	3-month administration of probiotic can restore the microbiota of CD patients similar to healthy children.
Harnett et al., 2016 [8].	A proprietary blend of 450 billion viable lyophilized bacteria (9 strains) known as the De Simone formulation, previously VSL#3.	12 weeks	Multicenter randomized Placebo-controlled trial	45 (*n =* 23 VSL#3, *n =* 22 placebo)	Microbial counts and comparison between baseline and end-of-study of predominant, pathogenic and opportunistic bacteria. Urinary metabolomics and fecal lactoferrin.	No significant changes in the gastrointestinal microbial counts in CD individuals with persistent symptoms over 12 weeks period.
Martinello et al., 2017 [62].	Yogurt with probiotic from PIA, Nova Petropolis-RS (undetermined microbial concentration).	30 days	Case-control study	14 CD patients, 17 healthy control	Fecal *bifidobacteria* concentration after consuming 100 g of yogurt in the morning.	Fecal *Bifidobacteria* count was higher in healthy patients compared to CD patients. Probiotic yogurt consumption increased the *Bifidobacteria* number in CD patients but not in healthy participants.
Pinto-Sanchez et al., 2017 [63]	*B. infantis Natren Life Start* super strain.	6 weeks	Double-blinded, randomized, placebo-controlled study	41 (*n =* 24 active no treatment CD, *n =* 12 active CD *B. NLS*, *n =* 5 GFD)	Determine mucosal expression of innate immune markers: number of macrophages, Paneth cells and α-defensin-5 expression by immunohistochemistry in duodenal biopsies.	Duodenal biopsies revealed that *B. infantis* NLS-SS decreased all the three markers in CD patients. However, the decrease in macrophage counts was higher in GFD.
Francavilla et al., 2019 [64].	A product containing five strains: *L. casei, L. plantarum, B. animalis* subsp. Lacti, *B. breve* Bbr8 LMG P-17501 and *B. breve* B110 LMG P-17500.	A 6-week treatment period, precede by 2-week run in period followed by a 6 week follow up phase for a total of 14 weeks.	Prospective, double- blind, randomized placebo-controlled parallel group study	109 (*n =* 54 probiotics, *n =* 55 placebo)	Determine if probiotics improve GI symptoms as assessed by IBS-SSS.	Probiotics significantly decreased the IBS-SSS and GSRS scores compared to the placebo, reduced IBS-type symptoms. Probiotics in CD patients on strict GFD diet modified the gut microbiota (increase the *Bifidobacteria*).
Primec et al., 2019 [65].	*Bifidobacterium breve* strains B632 and BRO3.	3 months.	Double-blinded, placebo-controlled study	40 CD children (*n =* 20 probiotics, *n =* 20 placebo), 16 healthy control	Evaluate the influence of probiotics on the fecal microbiome, SCFA and serum TNF-α.	*Verrucomicrobia, Paracubacteria* and some yet unknown phyla of bacteria and archaea showed a strong correlation to CD.
Uusitalo et al., 2019 [66].	*L. reuteri; L. rhamnosus*, and some unidentified.	Different time periods	Prospective study	6520	To study the association between the exposure of probiotics via dietary supplements or by infant formula since 1 year old for the development of CDA or CD.	Overall exposure of probiotics during the first year of age was not associated with CDA or CD. However, intake of probiotics via dietary supplements was associated with increased risk of CDA.

Celiac disease (CD), celiac disease autoimmunity (CDA), Irritable bowel syndrome (IBS), Irritative Bowel Syndrome severity scoring system (IBS-SSS), interleukin 10 (IL-10), Gastrointestinal (GI), Gastrointestinal Symptom Rating Scale (GSRS), Gluten-free diet (GFD), Natren life start (NLS) Short chain fatty acids (SCFA), Tumor Necrosis Factor alpha (TNF-α).
